# Dietary Omega-3 PUFA Intake in Patients with Chronic Kidney Disease: The Association with Vitamin D Deficiency, Intima–Media Thickness and Blood Pressure

**DOI:** 10.3390/jcm13185593

**Published:** 2024-09-20

**Authors:** Danijela Ristic-Medic, Marija Takic, Biljana Pokimica, Brankica Terzic, Milica Kojadinovic, Toplica Lepic, Slavica Radjen, Vesna Vucic

**Affiliations:** 1 Group for Nutritional Biochemistry and Dietology, Centre of Research Excellence in Nutrition and Metabolism, Institute for Medical Research, National Institute of Republic of Serbia, University of Belgrade, 11129 Belgrade, Serbia; biljana.pokimica@imi.bg.ac.rs (B.P.); milica.kojadinovic@imi.bg.ac.rs (M.K.); vesna.vucic@imi.bg.ac.rs (V.V.); 2 Group for Nutrition and Metabolism, Centre of Research Excellence in Nutrition and Metabolism, Institute for Medical Research, National Institute of Republic of Serbia, University of Belgrade, 11129 Belgrade, Serbia; marija.takic@imi.bg.ac.rs; 3 Clinic of Nephrology, Military Medical Academy, Faculty of Medicine, University of Defense, 11040 Belgrade, Serbia; brankica.terzic@gmail.com; 4 Clinic of Neurology, Military Medical Academy, Faculty of Medicine, University of Defense, 11040 Belgrade, Serbia; lepict@gmail.com; 5 Institute of Hygiene, Military Medical Academy, Faculty of Medicine, University of Defense, 11040 Belgrade, Serbia; slavicaradjen@gmail.com

**Keywords:** omega-3, carotid intima–media thickness, chronic kidney disease, cardiovascular risk, hemodialysis

## Abstract

**Background/Objectives:** Numerous risk factors associated with development of cardiovascular disease (CVD) have been unfavorably altered in patients with chronic kidney disease (CKD). Low omega-3 polyunsaturated fatty acid (PUFA) intake and vitamin D deficiency are potential cardiometabolic risk factors in patients with CKD. The aim of this study was to evaluate dietary intake and status of omega-3 PUFA and vitamin D in pre-dialysis and hemodialysis patients and to examine the association of dietary α-linolenic acid (ALA) and fish consumption with blood pressure and carotid intima–media thickness (C-IMT), representing a non-invasive marker of atherosclerosis in CKD patients. **Methods:** All 77 selected patients (36 pre-dialysis, 41 on hemodialysis) underwent standardized clinical, nutritional, and laboratory assessments. Repeated 24 h recalls were performed to assess dietary intake. The fatty acid profile was determined by gas–liquid chromatography. **Results:** Inadequate vitamin D intake and vitamin D status were found in 95% of patients. PUFA profiles did not differ between hemodialysis and pre-dialysis participants. Dietary intake of ALA was negatively correlated with systolic blood pressure (SBP) (*p* = 0.013), C-IMT (*p* = 0.002), serum CRP (*p* = 0.044), iPTH (*p* = 0.01), and 25(OH)D3 (*p* = 0.006). ALA intake of more than 0.23 g daily was linked with lower SBP (*p* = 0.001), serum 25(OH)D3 (*p* = 0.004), and C-IMT (*p* = 0.002). **Conclusions:** This study contributes to a better understanding of the relationship between dietary ALA intake and C-IMT in CKD. The results of this study could emphasize the significant role of the high prevalence of vitamin D deficiency and inadequate omega-3 PUFA intake and status regarding CVD health in CKD patients.

## 1. Introduction

Patients with chronic kidney disease (CKD) have an increased risk of developing cardiovascular disease (CVD) and mortality [1,2]. Therefore, early diagnosis of cardiovascular risk and the development of effective treatment strategies, including dietary management, to improve cardiovascular health in these patients are of paramount importance [3]. Ultrasound-guided determination of carotid artery intima–media thickness (C-IMT) is considered a reliable and non-invasive method recommended for CVD risk assessment in patients with CKD [4,5]. Hypertrophy of the intima and/or media layers of the carotid artery wall can be attributed to atherosclerosis [6]. Hypertension is a well-established risk factor for the progression of atherosclerosis as it impairs the function of the endothelium and exacerbates atherosclerotic plaques [7]. CKD is closely associated with hypertension [8].

The association between a lower intake of polyunsaturated fatty acids (PUFAs), especially linoleic acid (LA) and α-linolenic acid (ALA), and the occurrence of CKD in patients with type 2 diabetes has been observed [9]. According to data in the literature, intake of the long-chain omega-3 PUFAs eicosapentaenoic acid (EPA) and docosahexaenoic acid (DHA) could have a positive effect on elevated blood pressure [10,11]. However, the results in patients with kidney disease are inconsistent [12,13]; nonetheless, some data in the literature indicate that the effects of EPA and DHA supplementation on C-IMT could be beneficial in CKD patients on hemodialysis treatment [14]. The most important sources of long-chain omega-3 PUFAs are oily marine fish [15]. In addition, mammals can synthesize smaller amounts of both EPA and DHA from ALA, which is found in large quantities in plants (flaxseed, chia, walnuts, etc.) [16,17]. As components of cell membrane lipids, fatty acids influence their structure and function, and long-chain omega-3 fatty acids are substrates for anti-inflammatory mediators [18]. Circulating fatty acids are markers of the dietary intake and endogenous metabolism of these compounds. The levels of fatty acids in the erythrocyte membrane and plasma are longer- and shorter-term markers, respectively, for omega-3 intake [19]. A recent pooled analysis of prospective studies reported an inverse association between CKD risk and long-chain omega-3 PUFAs in the circulation [19]. Several authors demonstrated that omega-3 PUFA levels in the blood of dialysis patients were reduced compared to levels in healthy controls in their studies [20,21]; however, the reference risk range has not been established yet [20].

Vitamin D influences cardiovascular health due to a variety of effects, such as protective effects on cardiomyocytes and endothelial cells [22]. Vitamin D status in serum depends on synthesis in the skin after UV-B exposure, as well as on dietary intake and subsequent hydroxylation in the liver. The biologically active form of vitamin D is obtained in the kidney after the second hydroxylation step [23]. Vitamin D deficiency has been found in CKD patients before dialysis [24,25], and in patients undergoing dialysis, it occurs concomitantly with increased C-IMT [26].

Therefore, our study aimed to assess the dietary intake and status of omega-3 fatty acids and vitamin D in CKD patients and to investigate the relationship of ALA and fish consumption with blood pressure and C-IMT in renal failure.

## 2. Materials and Methods

### 2.1. Study Design

This cross-sectional study involved 77 patients with CKD (36 pre-dialysis and 41 hemodialysis patients) who were examined at the Department of Hemodialysis, Clinic of Nephrology, Military Medical Academy, Belgrade, Serbia. The diagnostic criterion for CKD is a glomerular filtration rate (GFR) of <60 mL/min per 1.73 m^2^ or a urinary albumin-to-creatinine ratio of >30 mg/g. The GFR rate was calculated using the following CKD-EPI creatinine equation [27]: eGFR = 142 × min (standardized S_cr_/k, 1)^α^ × max(standardized S_cr_/k, 1)^−1.200^ × 0.9938^Age^ × 1.012 [if female].

S_cr_ = serum creatinine in mg/dL; k = 0.7 (females) or 0.9 (males); α = −0.241 (female) or −0.302 (males); min (standardized S_cr_/k) = the minimum of S_cr_/k or 1; max (standardized S_cr_/k) = the maximum of S_cr_/k or 1.

All CKD patients had stage 3–5 CKD. The patients in the hemodialysis unit were dialyzed regularly, three times a week for 4 h. Blood flow ranged between 250 and 300 mL/min. while the dialysis flow rate was set at 500 mL/min. All patients were treated with highly permeable membranes. The exclusion criterion was the presence of any of the following diseases: acute myocardial infarction, acute infectious diseases within three months prior to recruitment, neurological or psychiatric problems, severe neoplastic diseases, and liver or lung diseases. The patients were not obese and did not exhibit severe malnutrition; their BMIs ranged from 20 to 30 kg/m^2^). After the initial eligibility assessment, subjects taking omega-3 and vitamin D3 supplements were excluded. All patients voluntarily signed written informed consent to participate in this study. This study was approved by the Ethical Review Board of the Military Medical Academy, Belgrade, Serbia (Approval Project No. 8/15-17) and was conducted in accordance with the standards and principles of the Declaration of Helsinki. All patients underwent a comprehensive evaluation including standardized clinical, nutritional, and laboratory assessment. Patients were categorized as “non-smokers” or “smokers” (past or current smokers). Diabetes mellitus was defined as fasting serum glucose ≥ 7.0 mmol/L (126 mg/dL) or ≥11.1 mmol/L (200 mg/dL) at 2 h postprandially or current treatment with hypoglycemic agents. Hyperlipidemia was defined as fasting serum triglyceride ≥ 1.69 mmol/L (150 mg/dL) and/or LDL cholesterol ≥ 3.62 mmol/L (140 mg/dL) or current treatment with hypolipidemic agents. The guidance for the correction and monitoring of vitamin D deficiency in patients with stage 3–4 CKD defines severe deficiency as serum 25-hydroxyvitamin D (25(OH)D) levels below 12 nmol/L, mild deficiency as levels between 12–39 nmol/L, and vitamin D insufficiency as levels of 40 nmol/L [28]. We set the target value of 25(OH)D at 50 nmol/L according to the UK NICE and US Endocrine Society [28,29].

### 2.2. Measurement of Anthropometric Parameters, Body Composition, and Blood Pressure

Assessment of anthropometric parameters included height, weight, mid-arm circumference (MAC), and waist circumference (WC). The measurements were taken on lightly clothed persons without shoes. The WC was measured from the midpoint between the lateral iliac crest and the lowest rib to an accuracy of 0.5 cm. A WC of more than 102 cm (men) or 88 cm (women) indicates significantly increased cardiometabolic risk. Body mass index (BMI) was calculated as weight (kg)/height (m)^2^. Height was measured to the nearest 0.5 cm using a stadiometer attached to the wall. Body weight, percentage of body fat, visceral fat area (VFA), and waist-to-hip ratio (WHR) were measured using an InBody720 body analyzer (Biospace Co., Ltd., Seoul, Republic of Korea). All participants were asked not to eat or drink for at least three hours before the measurement.

Blood pressure was measured twice after sitting for 5 min. Mean blood pressure was calculated from two separate measurements with a digital blood pressure monitor (OMRON^®^ Automatic Blood Pressure Monitor, model HEM-705CP, Vernon Hills, Illinois 60061), and the mean of these measurements was used. Hypertension was defined as systolic blood pressure (SP) ≥ 140 mmHg, diastolic blood pressure (DP) ≥ 90 mmHg, or current treatment with antihypertensive medication, according to the guidelines of The European Society of Cardiology [30].

### 2.3. Ultrasound Examination

Trained physicians and an experienced sonographer (T.L.) conducted the high-resolution ultrasonographic scanning and interpreted the results using ultrasound imaging equipment (Toshiba Aplio 500, Tokyo, Japan) equipped with a 6–12 MHz linear transducer. Both the left and right carotid arteries were evaluated at the levels of the common carotid artery (CCA), carotid bifurcation (BF), and internal carotid artery (ICA). The C-IMT was defined as the distance between the leading edge of the lumen–intima interface and the media–adventitia interface. Carotid plaque was defined as a focal zone penetrating at least 0.5 mm into the arterial lumen, >50% of the surrounding intima–media thickness or a thickness of 1.5 mm above the distance between the lumen–intima and media–adventitia interfaces. Overall C-IMT was measured according to the Mannheim consensus on carotid intima–media thickness and plaque (2004–2006–2011) [31]. The 10 mm long plaque-free segment at the posterior wall of the proximal CCA up to the carotid bifurcation was examined using special software for automatic interface detection. The mean C-IMT values were calculated for each segment of both carotid arteries. Some studies have indicated that a C-IMT < 0.8 mm is associated with normal, healthy individuals, whereas a C-IMT value of 1 mm or more is associated with atherosclerosis and a significantly higher risk of CVD across all age groups [32].

### 2.4. Assessment of Dietary Intake

The dietary intake assessment of our CKD patients was based on three repeated 24 h dietary recalls, based on participants’ subjective report for one month. Food and beverage intake was recorded for the dialysis day, the day after dialysis, and a weekend day for all. In the pre-dialysis patients, food consumption was recorded for two weekdays and one weekend day. The survey methods provided a better understanding of the patients’ eating habits during the week. Food quantities were estimated using common household measures, kitchen tools, and packaging information. A region-specific, validated Food Atlas with 135 items was used as an additional tool for estimating portion sizes. The dietary questionnaires were processed using Diet Assess & Plan, advanced nutritional software, accessed on 11 March 2023 [33]. Consumption data were converted into nutrient intake estimates according to the Serbian Food Composition Database (FCDB) as (EuroFIR FCDBs, https://www.eurofir.org/food-information/food-composition-databases/ accessed on 11 March 2023) [34] at the Institute for Medical Research. Adequacy assessment for vitamin D intake was based on European Food Safety Authority (EFSA) recommendation for the general population, 15 μg/day, which was derived from a target (25(OH)D) level of less than 50 nmol/L [35].

### 2.5. Analysis of Biochemical Parameters

All blood samples were collected after 12 h of fasting, and serum samples were analyzed by centrifugation after standing for 30 min at 4 °C. The following biochemical parameters in serum were determined: glucose, lipid status (triglycerides as well as total, HDL, and LDL cholesterol), urea, creatinine, potassium, C-reactive protein (CRP), hemoglobin (Hb), albumin, iron, insulin, HbA1c, c-peptide, 25-OH-D3 (a biomarker for vitamin D), and intact parathyroid hormone (iPTH). The values of all listed biochemical serum parameters were measured spectrophotometrically, with the exception of CRP, using the Siemens Dimension Rxl Max analyzer. The CRP value was determined using a turbidimetric immunoassay with the same analyzer. Samples were also obtained with the anticoagulant EDTA. These samples were immediately centrifuged at 4 °C, and the erythrocytes were isolated. The isolated erythrocytes were washed with a physiological solution, followed by a centrifugation step performed three times. They were stored at −80 °C and used to analyze the fatty acid profiles of erythrocytes.

### 2.6. Determination of Fatty Acid Profiles of Serum and Erythrocytes

Total serum lipids were extracted using a chloroform/methanol mixture (2:1, *v*/*v*) according to the method of Folch [36]. The total lipid content of erythrocytes was prepared using the method of Harth [37]. The phospholipid fractions of serum and erythrocytes were isolated by one-dimensional thin-layer chromatography (TLC). Briefly, the solvent system for TLC separation was hexane-diethyl-ether acetic acid (87:12:1 *v*/*v*), and it was performed using Silica Gel GF plates (C. Merck, Darmstadt, Germany). In the next step, methyl esters for gas chromatography were prepared by transesterification [36]. Afterwards, the samples were analyzed using Shimadzu GC 2014 chromatograph (Kyoto, Japan) with a flame ionization detector on an Rt × 2330 column (60 m × 0.25 mm ID, film thickness of 0.2 μm; RESTEK, Bellefonte, PA, USA) using the following temperature program: the initial oven temperature of 130 °C was held for 10 min and then increased at a rate of 3 °C/min until it reached the final temperature of 220 °C, which was held for 20 min. The fatty acid methyl esters in samples were identified by comparing peak retention times with those obtained for standard mixtures PUFA−2 and/or 37 FAMEs (Supelco, Bellefonte, PA, USA). The results are presented as calculated relative abundances of individual fatty acids expressed as a percentage of total identified fatty acids.

### 2.7. Statistical Analysis

The complete statistical analysis of the data was carried out using the statistical software package SPSS/21. The normality of the data was assessed using the Shapiro–Wilk test. Data are presented as the means ± standard deviations (SDs) if they followed a normal distribution and as medians and interquartile ranges if they followed a non-parametric distribution. Categorical data are described with numbers and percentages. To determine whether there were statistical differences between the pre-dialysis (Pre-D) and hemodialysis (HD) groups, the chi-square test was used for categorical data, the unpaired Student’s *t*-test for parametric numerical data, and the Mann–Whitney U-test for non-parametric numerical data. One-way ANOVA followed by the Tukey post hoc test was used to compare PUFA levels, biochemical parameters, C-IMT levels, and blood pressure among the different categories of ALA intake (classified as ≤0.23, 0.23–0.37, and >0.37). Spearman’s rank correlation was used to determine whether there was a statistically significant correlation between the intake of omega-3 PUFAs, and the numerical parameters measured. All analyses were estimated at a statistical significance level of *p* < 0.05.

## 3. Results

### 3.1. General, Anthropometric, Biochemical, and Clinical (Blood Pressure and C-IMT) Parameters and PUFA Status in CKD Patients

The demographic, anthropometric, and biochemical parameters of the CKD patients who participated in this study are shown in Table 1. A total of 78 CKD participants were divided into two groups according to dialysis status: 37 pre-dialysis (Pre-D) and 41 hemodialysis (HD) patients. In both groups, the proportion of men was higher, 73% in the Pre-D and 78% in the HD. The subjects in the HD group were younger than those in the Pre-D group (mean age 57 ± 14 and 64 ± 12 years, respectively). The proportion of people with smoking habits was higher in the HD group than in the Pre-D group (44% versus 24%). As expected, hypertension, dyslipidemia, and diabetes were common feature in patients with CKD (Table 1).

In the HD group, serum creatinine and iron levels were significantly (*p* < 0.001) higher, while albumin and urea levels were significantly (*p* < 0.05 and *p* < 0.001, respectively) lower. These are expected values as urea and creatinine are measured before dialysis and 80% of HD patients take iron supplements. HD patients had significantly lower HbA1c and insulin levels (*p* < 0.01) and higher levels of C-peptide, WHR, and % BF (*p* < 0.05). Further analysis among participants revealed significantly (*p* < 0.05) lower vitamin D levels and higher C-IMT (*p* < 0.01) in the HD group than in the Pre-D. There were no other significant differences in biochemical and anthropometric parameters and blood pressure between these two groups.

The PUFA profiles in the serum and the erythrocytes also did not differ significantly in patients undergoing HD treatment compared to Pre-D patients (Table 2). There was a tendency towards a higher proportion of ALA and a lower proportion of EPA + DHA in serum phospholipids in HD patients.

### 3.2. Dietary ALA and Total Omega-3 Fatty Acid Intake of CKD Patients

The group comparison based on food intake showed that HD patients had a significantly (*p* < 0.05) higher energy intake and dietary fat intake (g/day) than Pre-D patients (Table 3). However, no difference was found between the groups when dietary fats were expressed as % of energy. For dietary fats, HD participants reported a higher total intake of omega-3 PUFAs (*p* < 0.05). When analyzing individual dietary PUFAs, HD participants consumed higher amounts of ALA per day (*p* < 0.05).

**Figure 1:**
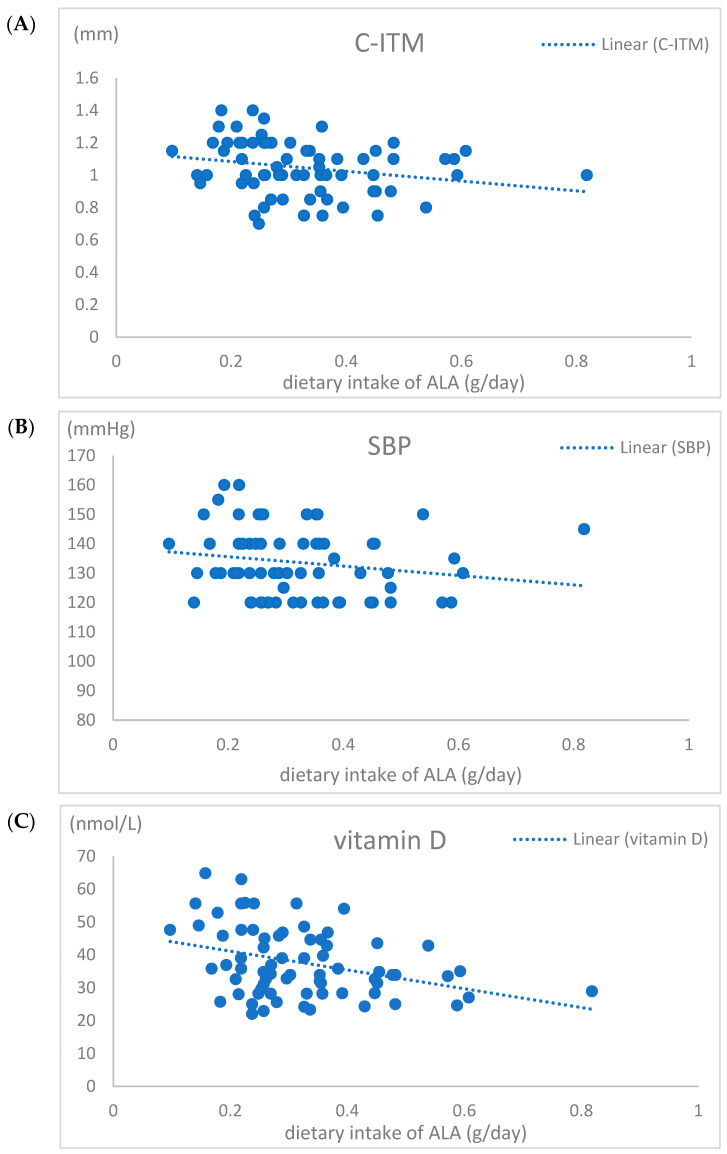
Relationship of dietary intake of α-linolenic acid with (**A**) carotid intima–media thickness (**C-ITM**), (**B**) systolic blood pressure (**SBP**), and (**C**) serum vitamin D concentration with added linear trendlines.

When the CKD patients were subdivided according to ALA intake (quantiles: first tertile ≤ 0.23, second tertile 0.23–0.37, third tertile > 0.37 g/day, number of participants 19, 33, and 19, respectively), it was found that LDL cholesterol (*p* < 0.045), SP (*p* < 0.013), serum 25(OH)D3 (*p* < 0.004), and C-IMT (*p* < 0.002) were significantly lower when ALA intake was more than 0.23 g/day (Table 4). Figure 1 presented key findings regarding the relationships between ALA intake and cardiovascular outcomes.

Only 18% of CKD patients reported eating fish regularly. There was no significant difference in the omega-6 or omega-3 PUFA profiles in serum or erythrocytes when we divided the CKD patients into groups according to tertiles of ALA intake.

### 3.3. Correlations of Biochemical, Anthropometric, and Clinical Parameters and PUFA Status with Estimated ALA and Fish Intake

Table 5 shows the correlation between estimated biochemical parameters and dietary intake of ALA in CKD patients. An inverse relationship was found between estimated ALA intake and serum CRP levels (*r* = −0.243, *p* = 0.044), iPTH (*r* = −0.303, *p* = 0.010), and 25(OH)D3 levels (*r* = −0.322, *p* = 0.006) (Table 5).

Dietary intake of ALA also negatively correlated with the anthropometric parameter MAC (*r* = −0.267, *p* = 0.025) and the clinical parameters SP (*r* = −0.316, *p* = 0.007) and C-IMT (*r* = −0.366, *p* = 0.002) (Table 6). In addition, fish consumption inversely correlated with C-IMT (*r* = −0.576, *p* = 0.042) (Table 6).

The percentages of omega-6 LA (*r* = 0.237, *p* = 0.032), omega-3 DPA (*r* = 0.449, *p* = 0.004), and total omega-3 (*r* = 0.317, *p* = 0.016) in serum phospholipids and EPA (*r* = 0.242, *p* = 0.042) in erythrocytes correlated directly with ALA intake (Table 7). Indirect correlations with dietary ALA intake were found for DGLA (*r* = −0.249, *p* = 0.037) and total omega-6 (*r* = −0.262, *p* = 0.027). Positive correlations were found for DHA (*r* = 0.621, *p* = 0.024) and total omega-3 in serum phospholipids (*r* = 0.599, *p* = 0.031) and for ALA in erythrocytes with fish intake (*r* = 0.729, *p* = 0.017) (Table 7). Inverse associations were also found between vitamin D concentration and C-IMT (*r* = −0.328, *p* = 0.005) and between SBP and EPA (*r* = −0.242, *p* = 0.042) as well as DGLA percentages in erythrocytes (*r* = −0.371, *p* = 0.001).

## 4. Discussion

To our knowledge, this is the first study to examine dietary ALA intake in CKD patients, considering both the PUFA profile and the relationship with C-IMT, CRP, and blood pressure under vitamin D deficiency conditions. It is noteworthy that all patients with chronic kidney failure examined had a low blood 25(OH)D3 concentration, which is a sensitive marker for vitamin D deficiency. Both vitamin D deficiency and inadequate dietary intake of omega-3 fatty acids were found in the studied patients. Inadequate vitamin D intake was found in up to 95% of CKD patients. Only 18% of patients confirmed their regular fish consumption, resulting in low EPA and DHA status.

In our study, the PUFA profiles in serum and erythrocytes showed no significant differences depending on dialysis status. It is noteworthy that hemodialysis patients consumed more omega-3 fatty acids than pre-dialysis patients, although their intake remained below the recommended levels. In addition, hemodialysis patients consumed less vitamin D, resulting in lower 25(OH)D3 levels that had a significant impact on their C-IMT values (*p* < 0.05). Patients who consumed more ALA in their diet appeared to have lower C-IMT, SP, and LDL cholesterol levels and lower concentrations of vitamin D in their blood. In addition, ALA intake was indirectly in correlation with the inflammatory marker CRP, iPTH levels, and SP. According to the obtained results regarding fish consumption, it could favorably change C-IMT in CKD patients. Correlations between fish consumption and DHA in serum phospholipids indicated associations between consumption and biomarker status. However, the findings regarding fish consumption were based on only 18% of respondents.

Vitamin D deficiency occurs more frequently in patients with CKD than in the general population [37]. In studies performed to date, 70–80% of CKD patients have been found to have vitamin D levels of less than 50 nmol/L. In our patient sample, 82% of patients had a 25(OH)D3 blood level below 50 nmol/L. All CKD patients in this study had a 25(OH)D3 blood level below 75 nmol/L. The samples were taken in the fall/winter. Patients had not taken vitamin D3 supplements for up to three months prior to measurement, although these baseline levels represent the recommendation for supplementation. Various foods fortified with vitamin D, such as bread, are not widely available on our market. The dietary recommendations for vitamin D intake are 10–20 μg/day, according to guidance [28,38]. However, the estimated average vitamin D intake in our patients was 2.2 μg/day in pre-dialysis patients and 1.8 μg/day in HD patients. The richest food source of vitamin D is fatty fish, followed by egg yolk, red meat, and liver. ALA is found in large quantities in the lipid fraction of some seeds and nuts (linseed, perilla, chia, and walnuts). The significant inverse relationship between dietary ALA intake and vitamin D status in our study is because these nutrients are present in different foods. Pre-D CKD patients have previously been found to have a higher incidence of vitamin D deficiency [24,25]. Our results suggest that vitamin D deficiency is exacerbated in hemodialysis patients. Karakas et al. [26] showed increased C-IMT in vitamin D-deficient patients undergoing dialysis. This is also consistent with our results. The 25(OH)D3 concentration in the blood is also indirectly related to C-IMT in our CKD patients.

Patients with CKD have increased cardiovascular morbidity and mortality [1,2]. The intake of omega-3 PUFAs, including those of both plant origin (ALA) and marine origin (EPA and DHA), could reduce the risk of cardiovascular disease [19,39] and have a positive effect on the cardiovascular health of CKD patients [40]. In this study, only 18% of participants consumed fish. Considering the current recommendation for ALA intake of 1.1 g/day for women and 1.6 g/day for men [41] and the fact that even higher amounts (>2 g/day) may be required for cardiovascular benefits [42], none of the subjects in this study consumed sufficient amounts of this plant fatty acid in their diet. The average intake was very low, 0.30 ± 0.10 g/day in the Pre-D group and 0.34 ± 0.15 g/day in the HD group, with the highest intake being 0.80 g/day. Recent studies have found an inverse relationship between the intake of omega-3 PUFAs, including ALA, and the prevalence of CKD [43], but there is little scientific data on the consumption of ALA in CKD patients. For instance, in a group of patients on hemodialysis, the reported dietary intake of ALA was 1.5 ± 1.0 g/day, and adequate dietary intake of omega-3 PUFAs was achieved in 33.3% of men and 30.6% of women [44], which is significantly higher than the levels found in our study. However, in a large cohort (n = 53,909), Bork et al. [45] emphasized the importance of the cardioprotective effect of ALA in participants with a very low intake of omega-3 PUFAs. The results of our study on the association between ALA intake and cardiovascular and cardiometabolic risk markers may therefore be of great interest for uncovering the role of ALA in the prevention of cardiovascular disease.

The results of a recent meta-analysis of 47 RCTs show that the consumption of ALA significantly reduces total cholesterol, LDL cholesterol, and triglyceride levels compared to a control diet [46]. When comparing tertiles of ALA intake, a significant difference in the effect on LDL cholesterol levels was found in our CKD patients, but there was no significant correlation between lipid parameters (triglycerides as well as total, HDL, and LDL cholesterol) and estimated dietary ALA intake. The progression of CKD leads to changes in lipid metabolism manifested by high concentrations of triglycerides, reduced HDL levels and increased levels of small, dense low-density lipoprotein (sdLDL) in the blood [47]. In this study, 39% of patients in the Pre-D group and 29% of patients in the HD group had hyperlipoproteinemic blood profiles. There was no difference in dietary ALA intake between the groups with and without dyslipidemia. The results obtained in this study in terms of measured blood lipidemia markers are consistent with some results of a recent meta-analysis by de Abreu et al. [48], which showed no beneficial effect of plant sources of ALA on lipid profiles in individuals with CKD. However, the observed positive effect on LDL cholesterol is probably significant, as the available literature data suggest that CKD patients have increased concentrations of atherogenic sdLDL particles in their blood.

A meta-analysis of 15 RCTs investigating the effect of flaxseed, a food source rich in ALA, on blood pressure showed a significant reduction in SP and DP [49]. The positive effect on blood pressure was also found in a recent RCT using walnuts [50] and a study by Hashimoto et al. [51] using perilla leaf powder as a source of ALA. When comparing the tertiles of ALA intake, a significant positive effect of ALA intake on SP was observed (*p* = 0.001). In addition, a significant inverse correlation was observed between ALA intake and SP.

However, there was no statistically significant difference when comparing the mean values of ALA intake between the groups with and without a diagnosis of hypertension, which may indirectly suggest that higher amounts of ALA are required to prevent the development of hypertension in CKD patients. The impact of low dietary ALA intake on blood pressure in CKD patients should be further investigated, as two major limitations of our study were that blood pressure was not monitored for 24 h and that some of the patients were receiving antihypertensive therapy.

In a meta-analysis of 25 RCTs conducted by Su et al. [52] found no significant effect of ALA supplementation on inflammatory markers in the blood, including CRP levels, although some studies showed its anti-inflammatory effect. However, in a recent meta-analysis, ALA was found to have a positive effect on lowering CRP levels in CKD patients [48]. In a previous study by our research group, a significant effect was obtained on the inflammatory markers IL-6, CRP, and TNF-α in r HD patients that consumed an ALA-rich seed mixture (3 g) for 12 weeks [53].The estimated ALA intake in the present study was very low, but even these very low dietary amounts have impact on CRP levels in CKD patients (comparison of tertiles of ALA intake and correlation between ALA intake and CRP levels). According to the data obtained, dietary ALA intake could have a strong positive effect on inflammation in CKD patients.

Few studies have examined the relationship between dietary intake or status of ALA and intima–media thickness as a marker of atherosclerosis, generally finding a beneficial effect of ALA. In a study by Ishikaza et al. [54] involving 1351 male subjects, an association was found between CKD and C-IMT. In addition, C-IMT was found to increase significantly with the stage of CKD [55]. Kajbaf et al. [14] investigated the effect of fish oil supplementation on C-IMT in CKD patients receiving hemodialysis treatment and concluded that fish oil may play a protective role in the progression of atherosclerosis in this patient group. As far as we know, there are no data in the literature on dietary ALA intake and its effects on IMT in CVD. Therefore, the main novel finding of this study is that dietary ALA intake (at very low levels, in patients with low fish oil intake) showed favorable effects on IMT, an important index of atherosclerosis (comparison of tertiles of ALA intake, *p* = 0.002; correlation between dietary ALA intake and C-IMT, *r* = −0.369, *p* = 0.002).

According to the available data in the literature as summarized in the meta-analysis by Sala et al. [39], ALA had little or no effect on the risk of type 2 diabetes (DMT2) and glycemic control. In this study, both diabetic and non-diabetic individuals were included (40% in the Pre-D group and 32% in the HD group). For this reason, the effect of ALA was not tested, as the Pre-D with/without DMT2 and HD with/without DMT2 groups included only a small number of subjects.

The type of fat consumed could be important for weight management. To date, several studies have shown an inverse relationship between ALA and measures of adiposity [56,57] and favorable outcomes in adiposity following supplementation with ALA-rich flaxseed [58]. CKD patients are at significant risk for malnutrition, even in the early stages of the disease, and maintaining a normal weight is critical for CKD prognosis [59]. According to the data obtained in this study, dietary ALA intake could influence the distribution of mass and have a significant effect on MAC (*r* = −0.267, *p* = 0.025) as well as WHR and VFA (comparison of tertiles of ALA intake). Van Duong et al. [60] recently reported that fat percentage and fat mass as well as MAC influence all-cause mortality in HD patients. The results of our study emphasize the importance of further research on the influence of ALA on body mass in CKD patients.

It is known that the consumption of fish and omega-3 PUFAs is beneficial for cardiometabolic health. A recent meta-analysis suggests that higher fish consumption is associated with a lower risk of coronary heart disease [10]. Several observational studies conducted in Western populations with a relatively low average intake of fish and seafood has shown that their higher dietary intake is associated with lower C-IMT [61]. Our patients also had low average fish consumption. Many health organizations and experts recommend that healthy adults should consume 250–500 mg/day of EPA + DHA. This can be achieved by eating two servings of oily fish per week [42]. The average consumption of fish and seafood was 83 g/day in consuming CKD patients (18%) and 9.9 g/day in all CKD patients. This study found a significant negative correlation between fish consumption and C-IMT in CKD patients. Therefore, a higher intake of omega-3 PUFAs could be a preventive dietary measure to prevent increase of C-IMT value related to the atherosclerosis process. The correlation between fish consumption and DHA in serum phospholipids in our study suggests that DHA could be a valuable biomarker associated with fish oil intake in CKD patients. Factors that determine the fatty acid profile should be further confirmed in larger studies, giving the fact that the fatty acid profile of patients undergoing hemodialysis could be a CVD risk marker [62].

Several limitations of this study should be considered. Firstly, the sample size is relatively small, which could reduce the statistical power of the observed relationships, and our findings should be confirmed and clarified by further larger-scale studies. We used one-way ANOVA but followed with post hoc test to explore the differences between groups and linear correlation analysis to explore potential relationships between tested parameters, but not regression analysis. Furthermore, there are numbers of confounders that could influence the findings the main being age, dietary intake of nutrients (such as SFA, salt, and sugars), glucose homeostasis, and weight status. So, our results should be confirmed and clarified by further studies on a larger scale. Due to the cross-sectional design of the study, the possibility of residual confounding cannot be excluded. Dietary intake of vitamin D and fatty acids was also estimated using self-reported, retrospective 24 h dietary recall. All methods for the assessment of dietary exposure have their inherent limitations 24 h dietary recall has inherent limitations for foods that are not consumed daily, such as fish. We also cannot determine whether there are differences in the protective effect of fish depending on the method of preparation (fried or not) or the origin (farmed or wild). Finally, no gender-specific considerations were made in the statistical analysis, as female participants were underrepresented in the study sample, accounting for only 17%.

## 5. Conclusions

In summary, due to the observed vitamin D deficiency in CKD patients and low omega-3 PUFA status, it is necessary to determine serum vitamin D levels together with estimated dietary intake as a standard for screening nutritional status. Therefore, nephrologists need to consider the nutritional management of renal disease in addition to the pharmacologic axis. This study contributes to a better understanding of the relationship between dietary omega-3 PUFA intake and C-IMT levels in CKD. ALA intake emerged as a factor that may influence C-IMT and SP in CKD patients. Our findings may have implications for CVD prevention and nutritional management in these patients. Although further investigations are needed, supplementation of vitamin D and omega-3 PUFAs in CKD patients may be beneficial in preventing and mitigating adverse health outcomes.

## Figures and Tables

**Table 1:** Characteristics of CKD patients.

Variable	Pre-D Group (n = 36)	HD Group (n = 41)
Age (years)	64 ± 11	57 ± 14 *
Male n (%)	27 (73)	32 (78)
Diabetes mellitus	40%	32%
Hypertension	36%	39%
Hyperlipidemia	39%	29%
Current smokers	24%	44%
BMI (kg/m^2^)	25.93 ± 5.54	24.56 ± 3.67
WHR (cm)	0.89 (0.71–0.21)	0.98 (0.76–1.34) *
WC (cm)		
Men	94 ± 10	97 ± 11
Women	87 ± 20	85 ± 13
MAC (cm)	26.86 ± 3.92	25.41 ± 4.22
BF (%)	25.55 ± 9.5	26.62 ± 7.62 *
VFA (cm^2^)	75 (23–218)	110 (32–241) *
Hg (g/L)	100 ± 13	100 ± 15
Urea (mmol/L)	26.94 ± 9.28	23.02 ± 5.45 *
Creatinine (μmol/L)	547 ± 177	915 ± 148 ***
Albumin (g/L)	44.20 ± 3.47	37.20 ± 3.27 ***
Total cholesterol (mmol/L)	4.19 ± 1.16	4.44 ± 0.93
TG (mmol/L)	1.47 ± 0.82	1.45 ± 0.82
HDL cholesterol(mmol/L)	1.04 (0.65–2.45)	1.00 (0.60–1.0)
TG/HDL	1.13 (0.40–2.36)	1.32 (0.29–7.07)
LDL cholesterol (mmol/L)	2.03 (1.07–6.04)	2.92 (1.30–4.54)
CRP (mg/L)	4.7 (0.98–33.1)	4.5 (2.03–24.5)
Iron (µmol/L)	9 (2–48)	14 (4–30) ***
Fasting glucose (mmol/L)	6.01 ± 1.79	5.46 ± 1.46
HbA_1_C (%)	6.15 ± 1.33	5.49 ± 0.92 **
C-peptide (nmol/L)	5.12 (0.05–19.60)	8.46 (1.1–17.29) *
Insulin (uIU/mL)	12.8 (5.20–89)	9.81 (1.60–44.60) *
iPTH (pg/mL)	35.6 (5.80–194)	45.51 (4.80–186)
Vitamin D (nmol/L)	42.80 (28.2–64.80)	33.90 (22–55.8) *
C-IMT (mm)	1.00 (0.75–1.30)	1.20 (0.70–1.55) **
SBP (mmHg)	133 ± 13	136 ± 11
DBP (mmHg)	88 ± 9	88 ± 7

Continuous variables are shown as the mean ± standard deviation for a normal distribution or the median (interquartile range) for a non-normal distribution. Abbreviations: Pre-D, pre-dialysis; HD, hemodialysis; BMI, body mass index; F, female; M, male; WHR; waist-to-hip ratio; WC, waist circumference; MAC, mid-arm circumference; BF, body fat; VFA, visceral fat area; Hg, hemoglobin; TG, Triglyceride; CRP, C-reactive protein; iPTH, intact parathyroid hormone; HbA_1_C, glycated hemoglobin; C-IMT, carotid intima–media thickness SBP, systolic blood pressure; DBP, diastolic blood pressure. * *p* ≤ 0.05; ** *p* ≤ 0.01; *** *p* ≤ 0.001 HD vs. Pre-D group.

**Table 2:** Serum and erythrocyte PUFA status in patients with CKD.

	Serum		Erythrocytes	
Fatty Acid %	Pre-D Group	HD Group	Pre-D Group	HD Group
18:2 n-6 (LA)	21.12 ± 3.83	21.95 ± 3.15	10.86 ± 1.33	11.20 ± 1.25
20:3 n-6 (DGLA)	2.79 ± 0.83	2.86 ± 0.64	2.22 ± 0.50	2.25 ± 0.50
20:4 n-6 (AA)	11.92 ± 3.13	11.78 ± 2.25	17.28 ± 2.13	17.35 ± 1.75
22:4 n-6	0.70 (0.35–1.41)	0.66 (0.23–1.73)	4.80 ± 1.01	4.75 ± 0.72
Σ Omega-6	36.58 ± 3.86	37.81 ± 4.63	35.16 ± 2.35	35.55 ± 2.07
18:3 n-3 (ALA)	0.09 (0.03–0.34)	0.11 (0.03–0.48)	0.13 (0.05–0.54)	0.13 (0.03–0.45)
20:5 n-3 (EPA)	0.21 (0.07–0.70)	0.22 (0.09–0.59)	0.7 (0.03–0.37)	0.10 (0.04–0.49)
22:5 n-3 (DPA n-3)	0.55 ± 0.13	0.60 ± 0.21	1.73 ± 0.34	1.88 ± 0.37
22:6 n-3 (DHA)	2.51 ± 0.80	2.26 ± 0.57	4.30 ± 1.12	4.24 ± 0.96
Σ Omega-3	3.41 ± 0.89	3.25 ± 0.74	6.36 (3.65–9.02)	6.61 (3.94–8.60)
Omega-6/omega-3	11.28 ± 2.47	12.25 ± 3.49	5.79 ± 1.15	5.73 ± 1.02
EPA + DHA	2.74 ± 0.85	2.51 ± 0.60	4.42 ± 1.10	4.36 ± 0.94
ΣPUFAs	39.99 ± 4.35	41.06 ± 4.68	41.47 ± 3.10	41.95 ± 2.84

Continuous variables are shown as the mean ± standard deviation for a normal distribution or the median (interquartile range) for a non-normal distribution. Abbreviations: Pre-D, pre-dialysis; HD, hemodialysis; LA, linoleic acid; DGLA, dihomo-gamma-linolenic acid; AA, arachidonic acid; ALA, α-linolenic acid; EPA, eicosapentaenoic acid; DPA, docosapentaenoic acid; DHA, docosahexaenoic acid; PUFAs, polyunsaturated fatty acids.

**Table 3:** Dietary intake of CKD patients.

Dietary Intake	Pre-D Group	HD Group
Energy (kcal)	1772 ± 157	1957 ± 297 *
Fat, % TE g/day	36.85 ± 7.24 71.5 (45.1–108)	37.35 ± 7.54 77.0 (38.9–138.4) *
Protein, % TE g/day	15.05 ± 4.55 66.57 ± 20.69	15.15 ± 3.24 74.52 ± 21.79
Carbohydrates, % TE g/day	48.09 ± 7.56 229.62 ± 36.84	47.50 ± 8.17 214.01 ± 44.95
Saturated FA, g/day	27.43 ± 8.04	30.99 ± 11.60
Monounsaturated FA, g/day	22.41 ± 5.17	25.22 ± 7.55
Polyunsaturated FA, g/day	16.17 ± 5.81	17.73 ± 10.84
Omega-3 FA, g/day	0.527 (0.339–1.140)	0.560 (0.242–1.743) *
Omega-6 FA, g/day	13.92 ± 5.21	13.65 ± 6.38
Linoleic FA (LA-18:2 n-6), g/day	12.41 ± 4.10	12.31 ± 5.79
Alfa-linolenic FA (ALA-18:3 n-3), g/day	0.30 ± 0.10	0.34 ± 0.15 *
Arachidonic acid FA (AA-20:4 n-6), g/day	0.08 ± 0.06	0.09 ± 0.06
Eicosapentaenoic FA (EPA-20:5 n-3), g/day	0.17 ± 0.12	0.18 ± 0.04
Docosahexaenoic FA (DHA-22:6 n-3), g/day	0.05 ± 0.11	0.04 ± 0.09
Vitamin D, μg/day	2.2 (0.57–5.75)	1.8 (0.8–5.4) *

Continuous variables are shown as the mean ± standard deviation for a normal distribution or the median (interquartile range) for a non-normal distribution. Abbreviations: Pre-D, pre-dialysis; HD, hemodialysis; LA, linoleic acid; ALA, α-linolenic acid; AA, arachidonic acid; EPA, eicosapentaenoic acid; DHA, docosahexaenoic acid; * *p* ≤ 0.05; HD vs. Pre-D.

**Table 4:** Characteristics of CKD patients according to estimated dietary ALA intake in g/day.

Variable	Tertile 1 ALA ≤ 0.23 g/Day	Tertile 2 ALA 0.23–0.37 g/Day	Tertile 3 ALA > 0.37 g/Day	*p*-Value
Age (years)	62 ± 13	61 ± 13	56 ± 13	0.053
n (%)	21 (27%)	35 (46%)	21 (27%)	
Diabetes mellitus	21%	27%	32%	
Hypertension	52%	36%	37%	
Hyperlipidemia	42%	30%	42%	
Current smokers	37%	41%	37%	
BMI (kg/m^2^)	25.01 ± 6.07	25.04 ± 3.88	24.47 ± 3.46	0.423
WHR (cm)	0.98 (0.71–1.21)	0.91(0.78–1.34) ^a^	0.94 (0.83–1.17) ^b^	0.019
MAC (cm)	24.83 ± 4.43	25.77 ± 4.26	27.84 ± 3.19	0.825
BF (%)	28.62 ± 8.31	24.94 ± 8.46	24.27 ± 8.41	0. 083
VFA (cm^2^)	103 (44–218)	76 (23–241) ^a^	84 (32–156) ^b^	0.049
Hg (g/L)	102 ± 18	99 ± 12	105 ± 13	0.638
Albumin (g/L)	39.32± 4.53	40.55 ± 5.27	40.68 ± 3.42	0.481
Total cholesterol (mmol/L)	4.36 ± 1.18	4.31 ± 0.98	4.31 ± 1.13	0.268
TG (mmol/L)	1.37 ± 0.04	1.60 ± 0.80	1.47 ± 0.70	0.581
HDL cholesterol (mmol/L)	1.08 (0.65–1.88)	0.98 (0.73–1.49)	0.98 (0.60–1.83)	0.760
TG/HDL	1.04 (0.29–1.88)	1.47 (0.5–7.06)	1.34 (0.44–3.22)	0.976
LDL cholesterol (mmol/L)	2.88 (1.41–6.04)	2.85 (1.57–4.54)	2.70 (1.07–4.30) ^b^	0.045
CRP (mg/L)	4.90 (2.53–33.10)	5.01 (0.98–24.50)	3.59 (1.49–7.96)	0.587
Iron (µmol/L)	12 (5–25)	11 (2–48)	14 (8–30) ^b^	0.001
iPTH (pg/mL)	46 (12–194)	42 (5–85)	36 (12–86)	0.094
Vitamin D (nmol/L)	45.8 (22.0–64.8)	36.9 (22.9–62.9) ^a^	33.5 (24.3–54.8) ^b^	0.004
C-IMT (mm)	1.20 (0.95 ± 1.40)	1.00 (0.70–1.55) ^a^	0.90 (0.7–1.20) ^b^	0.002
SBP (mmHg)	140 ± 12	130 ± 11 ^a^	128 ± 8 ^b^	0.013
DBP (mmHg)	90 ± 7	87 ± 8	85 ± 7	0.683

Continuous variables are shown as the mean ± standard deviation for a normal distribution or the median (interquartile range) for a non-normal distribution. Tukey post hoc test between-group analyses: ^a^ *p* ≤ 0.05 compared to tertile 1 ^b^ *p* ≤ 0.05 compared to tertile 2. ^a^ *p* ≤ 0.05 compared tertile 3 vs. tertile 2; Abbreviations: BMI, body mass index; WHR; waist-to-hip ratio; MAC, mid-arm circumference; BF, body fat; VFA, visceral fat area; Hg, hemoglobin; TG, Triglyceride; CRP, C-reactive protein; iPTH, intact parathyroid hormone; C-IMT, carotid intima–media thickness SBP, systolic blood pressure; DBP, diastolic blood pressure.

**Table 5:** Correlation between dietary ALA and fish intake with biochemical parameters.

	Hg	Glu	Urea	sCr	Alb	Tchol	LDL	HDL	TG	TG/ HDL	CRP	Iron	iPTH	Vit D
**Dietary ALA**														
***r***	0.100	−0.073	0.174	0.246	0.123	0.030	0.081	−0.054	0.135	0.186	−0.243	0.078	−0.303	−0.322
***p***	0.406	0.543	0.147	0.040	0.308	0.806	0.571	0.687	0.262	0.158	0.044	0.519	0.010	0.006
**Fish**														
***r***	0.130	−0.390	0.237	0.158	0.302	0.164	0.185	−0.280	0.079	0.193	−0.352	0.391	−0.005	−0.335
***p***	0.703	0.188	0.445	0.607	0.339	0.593	0.661	0.434	0.796	0.594	0.238	0.187	0.998	0.263

Abbreviations: ALA, α-linolenic acid; Hg, hemoglobin; Glu, glucose; Alb, albumin; sCr, serum creatinine; Tchol, total cholesterol; LDL, low-density lipoprotein; HDL, high-density lipoprotein; TG, triglycerides; CRP, C-reactive protein; iPTH, intact parathyroid hormone; Vit D, vitamin D.

**Table 6:** Correlation of dietary ALA and fish intake with anthropometric and clinical parameters.

	BMI	WHR	WC	MAC	%BF	VFA	SBP	DBP	C-IMT
**Dietary ALA**									
***r***	0.016	−0.053	−0.039	−0.267	−0.164	0.060	−0.316	−0.087	−0.366
***p***	0.895	0.660	0.744	0.025	0.172	0.619	0.007	0.471	0.002
**Fish**									
***r***	−0.469	−0.220	−0.421	−0.442	−0.156	−0.161	−0.415	−0.443	−0.576
***p***	0.106	0.469	0.152	0.151	0.610	0.569	0.159	0.130	0.042

Abbreviations: ALA, α-linolenic acid, BMI, body index; WHR, waist-to-hip ratio; WC, waist circumference; MAC, mid-arm circumference; BF, body fat; VFA, visceral fat area; C-IMT, carotid intima–media thickness; SBP, systolic blood pressure; DBP, diastolic blood pressure.

**Table 7:** Correlation of dietary ALA and fish intake with PUFA status in serum and erythrocytes.

				**Serum**						
	**LA**	**DGLA**	**AA**	**22:4n-6**	**Omega-6**	**ALA**	**EPA**	**DPA**	**DHA**	**Omega-3**
**Dietary ALA**										
***r***	0.237	−0.249	0.174	0.049	−0.262	0.087	0.005	0.449	0.088	0.317
***p***	0.032	0.037	0.147	0.300	0.027	0.589	0.977	0.004	0.589	0.016
**Fish**										
***r***	0.426	−0.060	−0.225	−0.280	−0.070	0.729	0.033	0.314	0.621	0.599
***p***	0.143	0.847	0.459	0.354	0.803	0.017	0.915	0.297	0.024	0.031
				**Erythrocytes**						
	**LA**	**DGLA**	**AA**	**22:4n-6**	**Omega-6**	**ALA**	**EPA**	**DPA**	**DHA**	**Omega-3**
**Dietary ALA**										
***r***	0.135	0142	0.186	0.016	−0,174	0.186	0.242	0.174	0.123	0.186
***p***	0.262	0.188	0.158	0.885	0.147	0.158	0.042	0.147	0.308	0.158
**Fish**										
***r***	−0.352	0.440	−0.066	0.115	−0,352	0.729.	0.187	0.322	0,113	0.165
***p***	0.239	0.133	0.831	0.707	0.239	0.017	0.541	0.284	0.704	0.590

Abbreviations: LA, linoleic acid; DGLA, dihomo-gamma-linolenic acid; AA, arachidonic acid; ALA- α linolenic acid; EPA, eicosapentaenoic acid; DPA, docosapentaenoic acid; DHA, docosahexaenoic acid; PUFA, polyunsaturated fatty acids.

## Data Availability

The data presented in this study are available on request from the corresponding author. The data are not publicly available for ethical reasons.
